# Comprehensive Review on Pancreatic Head Cancer: Pathogenesis, Diagnosis, and Treatment Challenges in the Quest for Improved Survival

**DOI:** 10.7759/cureus.54290

**Published:** 2024-02-16

**Authors:** Shreya Singh, Anupama Sawal

**Affiliations:** 1 Anatomy, Jawaharlal Nehru Medical College, Datta Meghe Institute of Higher Education and Research, Wardha, IND

**Keywords:** malignancy, diagnosis, surgery, screening, pancreatic head cancer

## Abstract

This comprehensive review explores the complexities surrounding pancreatic head cancer, a highly fatal and challenging-to-treat illness with a survival rate of less than five years. Despite being a major contributor to cancer-related deaths, pancreatic head malignancy often eludes early detection due to its posterior location and high metastatic potential. The review delves into the associated symptoms, including gastric outlet obstruction and obstructive jaundice, highlighting the impact on the patient's eligibility for surgery. Examining recent advancements, the article discusses fast-track surgery recovery programs and emerging immunotherapeutic approaches, acknowledging the unique challenges posed by the immunosuppressive environment of pancreatic head cancer. Additionally, the review elucidates the intricate relationship between pancreatic cancer and glucose levels, emphasizing the role of islets of Langerhans in insulin production.

The pathogenesis section explores lifestyle and genetic factors contributing to pancreatic head carcinoma, shedding light on risk factors such as smoking, obesity, diabetes, and hereditary predispositions. The extensive analysis of pancreatic cancer diagnosis methods encompasses imaging techniques, biopsies, and biomarkers, emphasizing the challenges posed by late-stage diagnoses. Addressing treatment modalities, the review emphasizes the significance of surgery, chemotherapy, radiotherapy, and targeted therapy. The intricate details of neoadjuvant, immunotherapy, and microbial therapy provide a comprehensive understanding of evolving treatment strategies. The review concludes by highlighting promising areas of research, including oncolytic viral therapy and gene editing technology, aiming to enhance the limited treatment options for this devastating disease.

## Introduction and background

Despite so much advancement in medical science, pancreatic head cancer remains one of the most fatal and incurable illnesses, with a survival rate of less than five years. After lung and colorectal cancer, it continues to be the most significant cause of mortality [1]. Due to increased incidence and mortality rates, pancreatic cancer is predicted to overtake several other malignancies as the second-leading cause of cancer-related death by 2030. Studies have shown that 80% of pancreatic malignancy is found in the head region and the rest of the 20% is found in the neck, body, and tail region. The posterior location and high tendency for metastasis of the cell's pancreatic head make the malignancy present in it difficult to diagnose in the initial stages, making it almost unresponsive to treatments in the later stages [2]. 

Patients with pancreatic head malignancy were seen to have gastric outlet obstruction (GOO), also known as pyloric obstruction in the stomach. The portal vein is also seen to be pressed, causing ascites. The most common problem in a patient with pancreatic head carcinoma is the obstruction of the posteriorly placed bile duct, which leads to obstructive jaundice in patients. These all lead to symptoms like difficulty eating, loss of appetite, paleness of skin, excessive weight loss, and vomiting [3]. Thus, these symptoms weaken the human body, making it not eligible for further surgery; only 20-30% of individuals who qualify for surgery have been reported, but they often experience early reappearance and death [4]. Therefore, patients who are not eligible for surgery anymore are advised to endoscopic placement of a metal stent to relieve the obstruction [5]. Fast-track or enhanced recovery after surgery (ERAS) programs have been developed in recent years to reduce the body's stress response to surgery and accelerate postoperative recovery. These fast-track programs include preoperative education, avoiding fluid overload, multimodal analgesia, early oral intake, and enforced mobilization. Pancreaticoduodenectomy (PD) fast-track programs are safe, are practical, and minimize problems. Recent studies have shown that the acquired immune privilege of pancreatic head cancer, fueled by an immunosuppressive milieu, inadequate T-cell infiltration, and a low mutational burden, is a critical factor in its lethality. Despite the ineffectiveness of immunotherapies such as checkpoint inhibition and modified T cells, increasing data indicates that orthogonal combinations of these and other strategies may make it possible to treat pancreatic cancer with immunotherapy [1,2].

Patients with pancreatic head carcinoma were also seen to have fluctuation in their glucose levels because the cancer prevents the islets of Langerhans from producing insulin, which is needed to maintain the glucose and other sugar levels in the body [6]. Some patients with pancreatic head carcinoma (less than 1%) were also found with "type 3c diabetes", a rare type of diabetes. Pancreatic head carcinoma is a rare disease whose root cause remains unknown. Technology and new techniques of endoscopic ultrasonography-guided gastrojejunostomy (EUS-GJ) are showing positive results and are comparatively less painful than other methods [7]. Preventive initiatives, especially primary prevention techniques, will benefit from understanding the underlying risk factors and how they interact to decrease exposure and identify people at risk of acquiring this commonly deadly cancer. Pancreatic cancer detection rates and the precursor lesions that precede it are increasing. A better knowledge of the risk factors and symptoms connected to this illness is vital for health professionals and the general public to be aware of potential preventative and early detection techniques.

## Review

Search methodology

**Figure 1:**
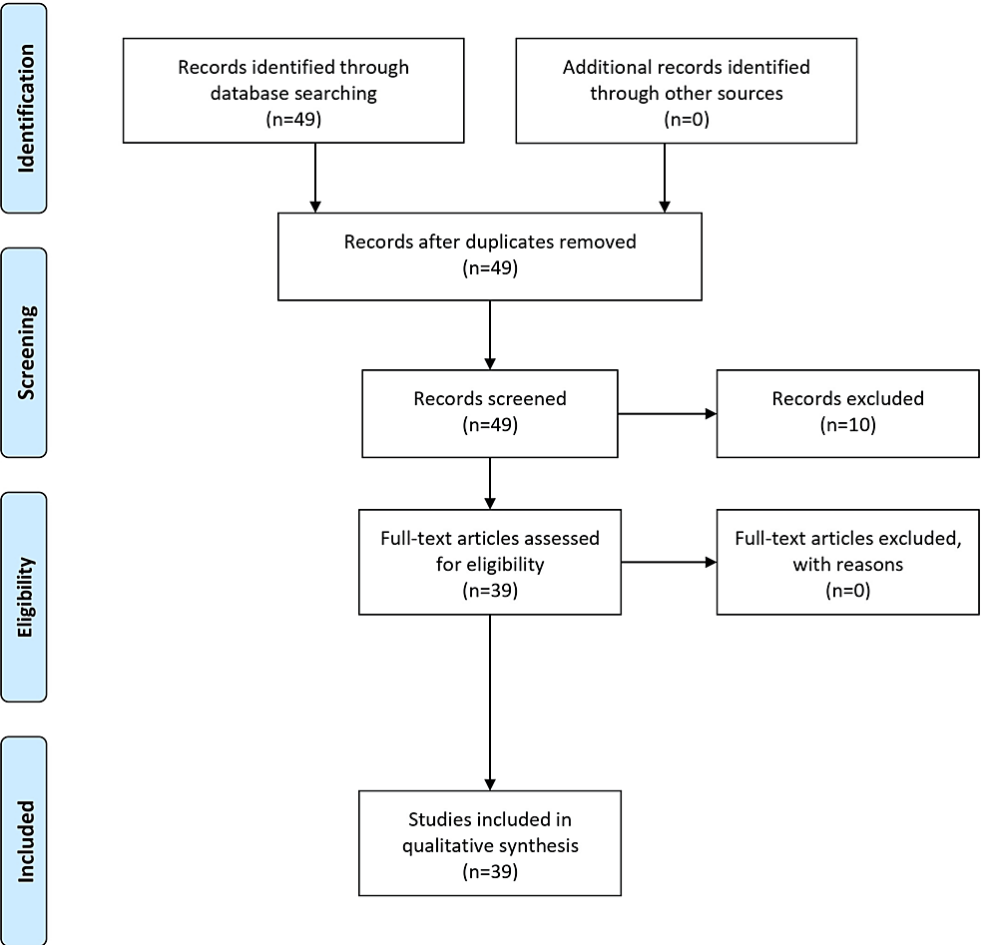
PRISMA flow diagram PRISMA: Preferred Reporting Items for Systematic Reviews and Meta-Analyses

The proposed comprehensive search methodology for a literature review on pancreatic head cancer involves leveraging major medical databases, cancer-specific repositories, and academic sources. Keyword selection covers diverse aspects, including risk factors, treatment options, and pathogenesis. Boolean operators refine searches, enhancing precision in results. Inclusion criteria prioritize recent, peer-reviewed studies, clinical trials, and systematic reviews, ensuring relevance and reliability. The article selection process involves meticulous screening of titles and abstracts, emphasizing studies on key aspects of pancreatic head cancer. Data extraction encompasses vital study details, including survival rates and emerging therapies. Quality assessment considers sample size, design, and statistical methods. Synthesizing findings emphasizes essential points, revealing gaps for future research. Reference management tools organize citations, ensuring clarity in sourcing. Continuous review through database updates keeps researchers abreast of the latest advancements. This robust methodology facilitates a comprehensive and up-to-date literature review on pancreatic head cancer. The Preferred Reporting Items for Systematic Reviews and Meta-Analyses (PRISMA) flow diagram is mentioned in Figure 1.

Pathogenesis

Till now, the specific pathogenic factor for pancreatic head carcinoma has not been identified. However, recent research has found that lifestyle and genetic modifications are the leading causes of this disease [8]. According to studies, smoking, obesity, diabetes, prolonged exposure to certain petrochemicals and pesticides, pancreatitis, and genetic causes increase the risk of pancreatic head carcinoma [8,9]. Obese adults have a 50-60% higher incidence of pancreatic head carcinoma than people with normal BMI [9]. Also, people with a high quartile of calorie intake have a 70% higher risk than those with a low-calorie intake [10]. Smoking cigarettes, cigars, and other tobacco products can also increase the risk of pancreatic head carcinoma [11]. Pancreatic fibrosis and inflammation brought on by the carcinogenic chemicals in tobacco-based products increase the risk of pancreatic head cancer [12]. Studies have shown that diabetic people are at higher risk of pancreatic head carcinoma than non-diabetic people [13,14].

About 80% of the patients initially show symptoms of either glucose intolerance or type 2 diabetes mellitus. This occurs because the cells that produce insulin (islets of Langerhans) are destroyed due to malignancy. In patients with pancreatic head carcinoma, glycated hemoglobin (HBA1c) and glucose levels are seen to be increased one month before diagnosis. HBA1c can be a potential biomarker for detecting pancreatic head cancer [14]. Genetics is the most common cause of pancreatic cancer, especially pancreatic head carcinoma [15]. Patients with a family history of pancreatic cancer sometimes develop comparable pancreatic malignancies than those who do not have a family history. Recent research has shown that heavy alcohol drinkers are at high risk of developing pancreatic cancer. Consumption of alcohol can lead to pancreatitis, which is the inflammation of the pancreas, which can lead to damage of the pancreas, leading to malignancy. Because of the many episodes of inflammation and healing, patients with hereditary pancreatitis have an elevated risk of pancreatic cancer. Research has shown that 80% of the patients have a family history of pancreatic head carcinoma and the majority of them had first-degree relatives who had already been given pancreatic cancer diagnosis [14,16]. According to studies, those with allergies live longer than those without and have a lower chance of developing cancer. Age, sex, ethnicity, blood type, and other factors can all raise the risk of developing pancreatic head cancer. Individuals with syndromes such as Lynch syndrome and Peutz-Jeghers syndrome are at higher risk of developing pancreatic head cancer. Older adults are often affected by pancreatic cancer. Patients are seldom diagnosed before the age of 30, and 90% of newly diagnosed patients are beyond the age of 55, with the majority being in their seventh or eighth decade of life.

Research has shown that men are more prone to pancreatic head carcinoma than women [17,18]. The danger of this disease is significantly increased with age; older people are more vulnerable to pancreatic head carcinoma than younger people. Most patients who received the diagnosis in recent years were over 60 years [19]. In the United States, it has been seen that African Americans are 50% more prone to pancreatic head carcinoma than Asian Americans [20]. According to a 2018 survey of pancreatic cancer patients, Europe had the most significant incidence rate of acute radiation syndrome (ARS), while Africa had the lowest [21]. Recent research has demonstrated that blood-type antigens can also influence pancreatic cancer risk. A, B, or AB blood types are more likely to develop pancreatic cancer than O blood types [22]. Age-adjusted incidence rates of pancreatic cancer are rising due to the increasing frequency of these risk factors in various parts of the world; however, the relative contribution of these risk factors differs across different areas due to differences in the underlying prevalence and preventative methods. While many risk factors can raise the likelihood of developing pancreatic head carcinoma, no single cause has been identified as directly responsible. Pancreatic head carcinoma symptoms are not obvious and usually develop over time. In 2020, around 57,600 new cases were detected, leading to 47,050 fatalities, according to the Surveillance, Epidemiology, and End Results (SEER) Program. This disease's early systemic dissemination and rapid local development are to blame for its dismal prognosis. Only 10-15% of individuals are known to survive this disease. Factors associated with pancreatic head carcinoma are described in Table 1.

**Table 1:** Factors associated with pancreatic head carcinoma

Risk factor	Increased risk	Mechanism
Pancreatic head carcinoma	NA	Lifestyle and genetic modifications
Smoking	Increased risk	Carcinogenic chemicals in tobacco-based products increase the risk of pancreatic head cancer
Obesity	50-60% higher incidence	NA
High-calorie intake	70% higher risk	NA
Diabetes	Higher risk	Destruction of insulin-producing cells (islets of Langerhans)
Family history	80% of patients have a family history	NA
Heavy alcohol consumption	High risk	Pancreatitis and damage to the pancreas
Age	Older adults are more affected	NA
Sex	Men are more prone	NA
Ethnicity	African Americans are 50% more prone	NA
Blood type	A, B, or AB blood types are more likely	NA

Diagnosis

The diagnosis of pancreatic head carcinoma is a very exacting process; this is due to the location of the pancreas. It sits deep in the abdomen and is surrounded by other internal organs, so tumours in the pancreas are not visible during the initial stages. Research has shown that 80-85% of tumours are diagnosed during the last stages, making them difficult to treat [23]. Early diagnosis of pancreatic head cancer depends upon the position of the tumour in the body.

In the general test, the skin, tongue, and eyes are examined to see if they are yellow, which can indicate obstructive jaundice. Obstructive jaundice can be due to blockage of the bile duct due to the tumour in the head of the pancreas, leading to pale skin and yellow sclera [24]. There is also an increase in bilirubin concentration seen in patients due to blockage of the bile duct. A biopsy helps in the definite diagnosis of any type of cancer, including pancreatic head carcinoma. It can be done by either fine needle aspiration (FNA), in which a fine needle is inserted in the pancreas and cells are suctioned out and studied in the laboratory, or through core needle biopsy, where extensive tissue is taken instead of cells. If a non-suspicious cyst has been found, there should be immediate screening. Due to advancements in science, new biomarkers that help in early cancer detection have been found. Till now, few biomarkers have been found that help in the early prognosis of pancreatic head carcinomas, such as microRNAs, carcinoembryonic antigen (CEA), CA125, CA242, CA19-9, and k-RAS gene mutation [25]. The US Food and Drug Administration has approved serum cancer antigen (CA19-9) as the most accurate biomarker for detecting pancreatic head carcinoma [26].

The level of CA19-9 is often increased in people with pancreatic head carcinoma. Because CA19-9 has a sensitivity of 68% for up to one year and 53% for up to two years before diagnosis, with a 95% specificity, it offers intriguing sensitivities for diagnosing preclinical pancreatic cancer. Similarly, CA125 has similar sensitivities. Since the concentration of CA125 was elevated in around 20% of CA19-9-negative patients, the combination of CA19-9 and CA125 enhanced sensitivity. Long-term survival is linked to baseline levels of CA19-9 that are normal. CA19-9 might be utilized as a promoter to assess the biology of pancreatic cancer. Through protein glycosylation, binding to E-selectin, bolstering angiogenesis, and modulating the immune response, CA19-9 can hasten the growth of pancreatic cancer. For evaluating the poor prognosis in pancreatic head carcinoma, biomarkers such as IL- 10, IL- 8, IL-6, and B7-H4 are used [27]. Although many biomarkers have been discovered recently, accurate biomarkers still exist to treat this disease [28]. Pancreatic head carcinoma is usually not visible during the early stages. That's why population-based screening is not suggested for this type of disease [29]. Individuals with a history of pancreatic cancer in their family are recommended for early screening [30,31]. Some of the best screening methods are magnetic resonance imaging (MRI), EUS, endoscopic retrograde cholangiopancreatography (ERCP), computed tomography (CT) scan, etc. The pancreatic juice can also be used for the diagnosis of this cancer. Pancreatic juice from patients with pancreatic intraepithelial neoplasia (PanIN) 2-3, moderate- and high-grade intraductal papillary mucinous neoplasm (IPMN), and invasive malignancy were discovered to include mutant P53.

Magnetic resonance cholangiopancreatography (MRCP) is a specialized type of MRI used to examine pancreatic tumours and blockage [32] accurately. EUS has been more precise in identifying cancer than all other screening methods [33]. People at a higher risk of developing pancreatic head cancer are advised for pancreatic cancer screening utilizing a combination of EUS and MRI/MRCP by the International Cancer of the Pancreas Screening Consortium group; this combination is very effective in identifying the tumour in the pancreas [34]. ERCP is used to identify any cancer in the head region of the pancreas and place bile duct stents if any tumour has been found. Recent studies have found that the efficiency of ERCP can be improved if aspiration cytology and brushing cytology are combined [35]. CT scan is used to pinpoint the precise position and size of the tumour. The American Society of Clinical Oncology has suggested that patients with pancreatic head cancer should undergo abdominal, pelvic, and chest CT scans every two to three months. MRCP is often saved for unclear patients and is a second-line technique for suspected pancreatic cancer. When identifying pancreatic cancer, CT and MRI have sensitivity levels of up to 96% and 93.5%, respectively. When employed as a one-time screening modality, secretin-enhanced MRI and MRCP have been demonstrated to have a good to excellent concordance with findings from EUS and avoid the danger of ionizing radiation. A recent study has found that patients diagnosed with pancreatic head carcinoma must be interpreted at least 18 times during treatment [36]. A significant amount of work is being done to provide new early detection screening tests and enhance the capacity of existing technologies to detect pancreatic cancer in its early stages.

Treatment

As pancreatic head cancer is often discovered in advanced stages, early treatment is challenging. The treatment procedure includes surgery, immunotherapy, radiotherapy, chemotherapy, targeted therapy, and microbial therapy. Surgery is the most efficient method of treating pancreatic head carcinoma. It could raise the likelihood of the patient surviving. The most excellent surgical procedure for pancreatic head carcinoma is PD or the Whipple procedure. Patients who have undergone PD depend on radical R0 resection [37]. Around 20-86% of patients cannot achieve R0 resection [38]. Still, the longevity of the patient's life can be increased by R1 resection.

In PD, the head of the pancreas, the first part of the small intestine, the gallbladder, and the bile duct are removed. Pancreatic cancer is known to be an immunosuppressive disease; hence, till now, there is no immunotherapy authorized for pancreatic head carcinoma. Chemotherapy is known to suppress the immune response, but patients with pancreatic head cancer have not experienced such a shift in response [35]. Studies examining the effects of neoadjuvant therapy have been carried out in patients with resectable or marginally resectable illness. Preoperative treatment may be more successful than postoperative therapy since the resected tumour bed is linked with poor drug delivery and limited sensitivity to radiation due to diminished oxygenation. It also increases the possibility of delivering full-dose chemotherapy if administered before surgery. In recent research, mass spectrometry of metabolites from cancer tissue revealed elevated levels of certain metabolic by-products in patients with early-stage pancreatic adenocarcinoma relative to controls. However, there was a discrepancy between the amounts found in plasma samples and cancer tissue, indicating that more research is necessary if a blood-based biomarker is to be created. Numerous clinical trials have examined the effect of neoadjuvant chemo(radio)therapy to enhance local control and, ultimately, patient survival. Several promising targeted treatments for pancreatic head cancer exist depending on the disease stage. The elimination of micrometastases and shrinking of the underlying tumour are two potential benefits of neoadjuvant therapy, and both of these outcomes are linked to a lower risk of cancer recurrence. Localized tumours have three classifications: locally progressed, borderline resectable, and resectable. Adjuvant chemotherapy is the standard of care for resectable tumours with limited vascular penetration and no distant metastases.

Radiotherapy is seen to be very useful in patients with localized pancreatic cancer [1,32]. External beam radiation therapy is the most used for pancreatic head carcinoma. Still, this therapy causes a lot of damage to other cells and typically involves a set number of treatments spread out over time. Brachytherapy is a form of internal radiation therapy intended to eradicate the tumour and the cancer cells surrounding it without damaging other healthy cells, unlike external beam radiation therapy. External beam radiation therapy and brachytherapy are occasionally combined for treatment [34]. Chemotherapy is the most crucial part of the treatment of pancreatic head carcinoma. A patient undergoing chemotherapy may be given one medication at a time or a combination of pills.

Drugs like gemcitabine and capecitabine or modified leucovorin, 5-fluorouracil, irinotecan, and oxaliplatin are given for six months after R0 resection. Gemcitabine combined with 54 Gy is given to patients with localized malignancy in the pancreas. Even though chemotherapy is one of the most effective cancer treatments, it has many side effects, such as poor appetite, hair loss, abdominal pain, nausea, gastrointestinal problems, vomiting, depression, and lack of energy [35]. Although cancer patients frequently experience discomfort from chemotherapy, patients with pancreatic head carcinoma suffer unbearable pain before chemotherapy compared to overall pain [36]. Partial surgical excision of the afflicted pancreatic area is the first step in the treatment protocol. In patients with locally advanced pancreatic cancer, the Eastern Cooperative Oncology Group (ECOG) 4201 study evaluated the effects of chemotherapy and chemoradiotherapy. The median survival for chemotherapy with gemcitabine was 9.2 months, whereas the median survival for chemoradiotherapy was 11.1 months. Immunotherapy is said to be a very effective treatment for pancreatic cancer. Cancer immunotherapy to stimulate the immune system against tumours has taken up a small portion of treatment choices. A paradigm change in the treatment of metastasis has resulted from targeting immunological checkpoint molecules through the suppression of cytotoxic T-lymphocyte-associated antigen 4 (CTLA-4), programmed cell death protein-1 (PD-1), and programmed cell death protein ligand-1 (PD-L1) using monoclonal antibodies. Treatment response previously stated genetic mutations or phenotypes and the patient's general condition significantly treat nonresectable and metastatic illness. Like with localized disease, the usual first-line therapy in most instances is gemcitabine or FOLFIRINOX (leucovorin calcium (folinic acid), fluorouracil, irinotecan hydrochloride, and oxaliplatin) in combination with albumin-bound (nab) paclitaxel, and for the patient who is capable of the second line of treatment, combination treatment like gemcitabine and FOLFIRINOX can be effective.

Targeted therapy is a type of treatment that targets the cancer cells and limits the spread of growth without damaging the healthy cells. Pembrolizumab, olaparib, and erlotinib are some drugs approved by the Food and Drug Administration to treat pancreatic head carcinoma. PEGPH20 and CKAP4 are new targets for treating pancreatic head carcinoma by targeted therapy [34]. In microbial therapy, groups of bacteria like *Proteus*, *Pseudomonas*, *Caulobacter*, and *Listeria* persuade the immune response and activate some immune cells that recognize and destroy the tumours [37]. Although there has been a lot of new research in treating this disease, we still do not know the actual treatment for this dreadful disease. The restriction in current treatment motivates further invention. Oncolytic viral therapy and gene editing technology are some research areas that have an optimistic future [38,39]. The treatment aspect of pancreatic head cancer is described in Table 2.

**Table 2:** Treatment aspect of pancreatic head cancer PD: pancreaticoduodenectomy

Treatment	Description	Side effects	Drugs
Surgery	Most efficient method	Raises survival chance	PD or Whipple procedure
Immunotherapy	No approved therapy yet	Suppresses immune response	
Radiotherapy	Useful for localized cancer	Damages other cells	External beam radiation therapy, brachytherapy
Chemotherapy	A most crucial part of treatment	Many side effects	Gemcitabine, capecitabine, 5-FU, irinotecan, oxaliplatin
Targeted therapy	Targets cancer cells	Pembrolizumab, olaparib, and erlotinib	PEGPH20, CKAP4
Microbial therapy	Uses bacteria to activate immune response		*Proteus*, *Pseudomonas*, *Caulobacter*, *Listeria*

## Conclusions

Pancreatic head cancer remains a formidable challenge in the realm of medical science, with a dismal survival rate and limited treatment options. The complexity of its pathogenesis, late-stage diagnosis, and limited eligibility for surgery contribute to its high mortality rates. The review underscores the importance of understanding risk factors, including lifestyle, genetics, and comorbidities, and the significance of early detection through evolving diagnostic techniques. While surgical interventions like PD offer a glimmer of hope, the overall effectiveness of available treatments, including chemotherapy, radiotherapy, and targeted therapy, is constrained. The emerging field of immunotherapy shows promise, but challenges persist due to the immunosuppressive nature of pancreatic cancer. The review emphasizes the need for continuous research into novel therapies, including microbial therapy and gene editing technologies, to improve treatment outcomes. Ultimately, a comprehensive approach involving early detection, personalized treatment strategies, and ongoing advancements in medical science is crucial to addressing the profound impact of pancreatic head cancer on patient outcomes.
